# Supplementary material for: A Comparative Characterization and Expression Profiling Analysis of Fructokinase and Fructokinase-like Genes: Exploring Their Roles in Cucumber Development and Chlorophyll Biosynthesis
Source: Int J Mol Sci. 2022 Nov 17;23(22):14260. doi: 10.3390/ijms232214260 (PMC9698557; doi:10.3390/ijms232214260)
Supplement: Supplementary file 1 [file ijms-23-14260-s001.zip › Table S2.pdf]

Table S2. Comparison of the characterization of *CsFRK*s and *CsFLN*s.

Gene name	Length of amino acids	Molecular weight (kD)	Isoelectric point
CsFRK1	384	41.18	5.69
CsFRK2	348	37.67	5.31
CsFRK3	331	35.64	5.62
CsFLN1	511	58.11	6.59
CsFLN2	588	65.90	5.90
