# Supplementary material for: A Comparative Characterization and Expression Profiling Analysis of Fructokinase and Fructokinase-like Genes: Exploring Their Roles in Cucumber Development and Chlorophyll Biosynthesis
Source: Int J Mol Sci. 2022 Nov 17;23(22):14260. doi: 10.3390/ijms232214260 (PMC9698557; doi:10.3390/ijms232214260)
Supplement: Supplementary file 1 [file ijms-23-14260-s001.zip › Table S3.pdf]

Table S3. List of corresponding relationship of *CsFRK*s and *CsFLN*s in two cucumber genomes.

Gene name	Gene ID in cucumber 9930 V2 genome	Gene ID in cucumber 9930 V2 genome	Identity
CsFRK1	Csa3G345390	CsaV3_3G021590	100%
CsFRK2	Csa5G172290	CsaV3_5G005580	100%
CsFRK3	Csa6G080910	CsaV3_6G006740	100%
CsFLN1	Csa3G732620	CsaV3_3G034260	100%
CsFLN2	Csa4G571770	CsaV3_4G031430	100%
