# Supplementary figures and images for: A Comparative Characterization and Expression Profiling Analysis of Fructokinase and Fructokinase-like Genes: Exploring Their Roles in Cucumber Development and Chlorophyll Biosynthesis
Source: Int J Mol Sci. 2022 Nov 17;23(22):14260. doi: 10.3390/ijms232214260 (PMC9698557; doi:10.3390/ijms232214260)

**Chr1****Chr2****Chr3****Chr4****Chr5****Chr6****Chr7**

0

5

10

15

20

25

30

35

40

45 (Mb)

**CsFRK2****CsFRK3****CsFRK1****CsFLN2****CsFLN1**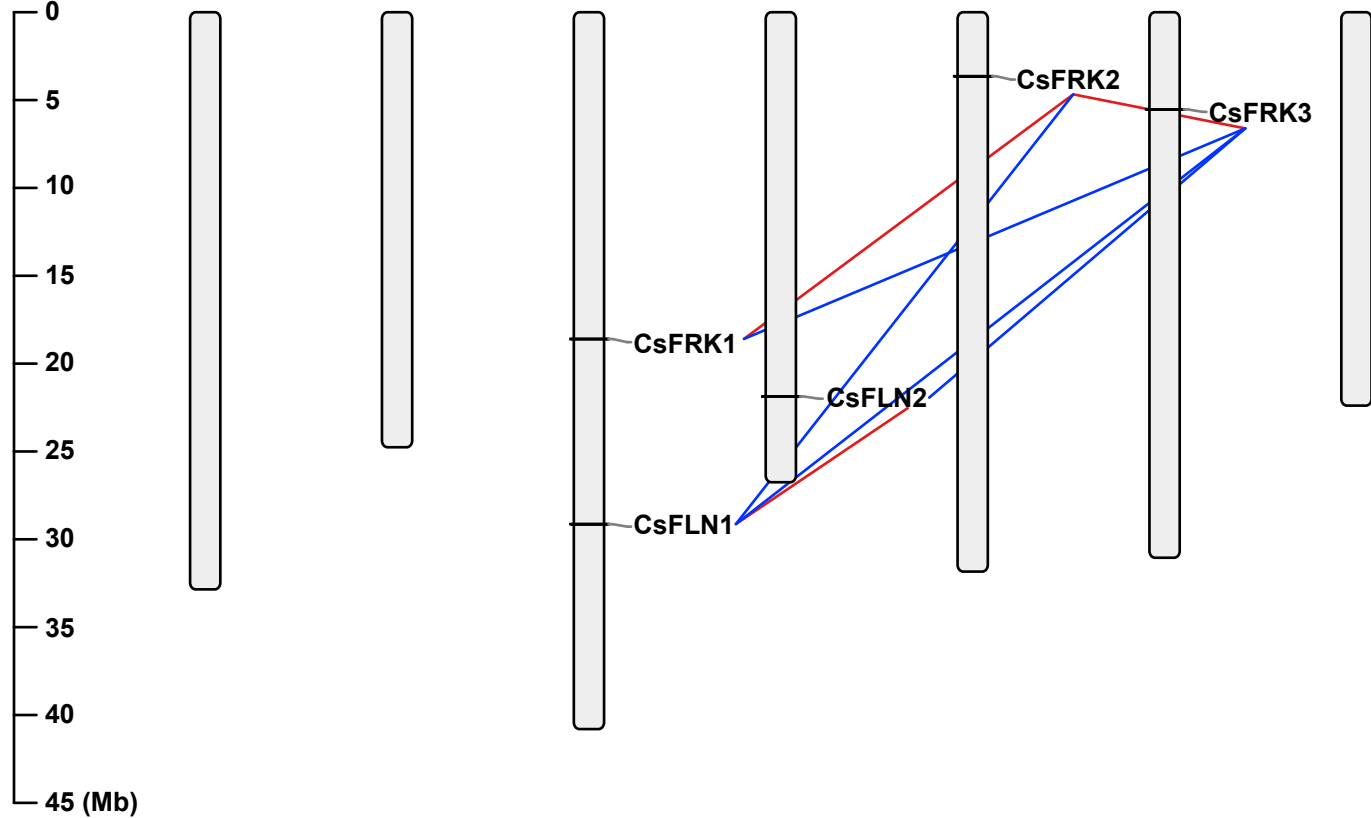

Supplement: Supplementary file 1 [file ijms-23-14260-s001.zip › Figure S1.pdf]
